# No association between proton pump inhibitor use and ALS risk: a nationwide nested case–control study

**DOI:** 10.1038/s41598-020-70373-8

**Published:** 2020-08-07

**Authors:** Hakan Cetin, Jiangwei Sun, Catarina Almqvist, Berthold Reichardt, Matthias Tomschik, Fritz Zimprich, Fang Fang, Caroline Ingre

**Affiliations:** 1 Department of Neurology, Medical University of Vienna, Vienna, Austria; 2 Institute of Environmental Medicine, Karolinska Institutet, Box 210, 17177 Stockholm, Sweden; 3 Department of Medical Epidemiology and Biostatistics, Karolinska Institutet, Stockholm, Sweden; 4 Astrid Lindgren Children’s Hospital, Karolinska University Hospital, Stockholm, Sweden; 5 Austrian Social Health Insurance Fund AT, Eisenstadt, Austria; 6 Department of Clinical Neuroscience, Karolinska Institutet, Stockholm, Sweden

**Keywords:** Motor neuron disease, Epidemiology

## Abstract

The use of proton pump inhibitors (PPIs) has been proposed as a potential risk factor for neurodegenerative diseases, but little is known regarding its role in amyotrophic lateral sclerosis (ALS). We therefore aimed to assess the association of PPI use with the subsequent risk of ALS, and performed a register-based nationwide nested case–control study, including 2,484 ALS cases diagnosed during July 2006–December 2013 in Sweden and 10 population controls per case that were individually matched to the case by sex, age, and area of residence. Dispenses and cumulative defined daily doses (cDDDs) of PPIs were extracted from the Swedish Prescribed Drug Register. The association of PPI use with the risk of ALS was assessed using conditional logistic regression, after applying different lag windows to avoid reverse causation. ALS patients were more likely to be dispensed with PPIs before diagnosis than controls. However, previous PPI use was not associated with an increased risk of ALS (OR = 1.08, 95% CI 0.97–1.19), and there was no dose–response relationship between cDDDs of PPIs and ALS risk (p = 0.0874), after excluding dispenses during the year before ALS diagnosis. The results were similar after excluding dispenses during the 2 or 3 years before ALS diagnosis.

## Introduction

Amyotrophic lateral sclerosis (ALS) is a rapidly progressing neurodegenerative disease characterised by limb and bulbar muscle weakness ultimately leading to respiratory failure and death^1^. Disease pathology is based on a multifactorial aetiology with a complex interplay of genetic and environmental risk factors^2^, some of which have also been reported in other neurodegenerative diseases^3,4^.

An increasing body of evidence based on experimental data suggests that lysosomal dysfunction results in defective protein clearance and plays a crucial role for the intracellular protein deposition characteristically found in neurodegenerative diseases including Parkinson’s disease^5^, Alzheimer’s dementia^6,7^ and ALS^8,9^. Those findings have paved the way for novel therapeutic strategies targeting pathways involved in the luminal acidification of lysosomes^10^. Proton pump inhibitors (PPIs), a widely prescribed drug class to reduce gastric acid and treat gastroesophageal reflux disease, pass the blood brain barrier^11,12^ and were shown to impair autophagy and lysosomal acidification by inhibition of vacuolar H^+^ ATPases, thereby hampering protein degradation and potentially contributing to disease pathology in neurodegenerative diseases^10,13,14^. Exposure to PPIs may therefore constitute a risk factor for proteinopathic neurodegenerative disease such as Parkinson’s disease, Alzheimer’s dementia and ALS. In fact, observational studies found evidence for an association between PPI use and an increased risk to develop Parkinson’s disease^15^ and dementia^16,17^, but no studies have yet assessed the link between exposure to PPIs and ALS risk.

The aim of this nationwide nested case–control study utilising several Swedish national health registers was to test the hypothesis that PPIs constitute a risk factor for ALS. As PPIs are one of the most widely prescribed drugs worldwide^18^ and ALS is one of the most devastating diseases to date, a small risk increment for ALS in relation to PPI use would still lead to significant public health implications.

## Results

A total of 2,484 cases with a new diagnosis of ALS and 24,840 matched controls were identified during the study period. Among cases, 1,674 (67%) were identified by their first ALS related hospital visit as outpatients, whereas 810 (33%) were identified by their first ALS related hospital visit as inpatients. In both cases and controls, the mean age at the index date was 68.6 years and men accounted for 56.7% of the study sample (Table 1). ALS patients were more likely to be dispensed with PPIs before the index date (28.3% among cases and 24.0% among controls, *p* < 0.0001, Chi-square test), corresponding to a significant ALS risk increment associated with PPI use (OR = 1.26, 95% CI 1.15–1.39). However, after exclusion of PPI dispenses in the first year before the index date from the analysis (i.e. including a lag window of 1 year) the difference diminished and was no longer statistically significant (21.7% among cases and 20.5% among controls, *p* = 0.0914, Chi-square test). The cumulative exposure to PPIs was higher among cases than controls (cDDDs of 515 ± 674 among cases and 504 ± 674 among controls) after including the lag window of 1 year, but the difference was again not statistically significant (*p* = 0.4380, T-test). Any PPI use before the index date was not associated with an increased risk of ALS after including a lag window of 1 year (OR = 1.08, 95% CI 0.97–1.19), and there was no dose–response relationship between cDDDs of PPIs and ALS risk (*p* = 0.0874) (Table 2). The association did not vary by age or sex and the results were similar using lag windows of 2 or 3 years.

**Table 1:** Characteristics of ALS patients and the matched controls, a nested case–control study in Sweden, 2006–2013.

Characteristics	Patients with ALS			Controls		
	Total	Men	Women	Total	Men	Women
N (%)	2,484	1,408 (56.7)	1,076 (43.3)	24,840	14,080 (56.7)	10,760 (43.3)
Age at index date in years (mean ± SD)*	68.6 ± 11.7	67.9 ± 11.6	69.7 ± 11.8	68.6 ± 11.7	67.9 ± 11.6	69.7 ± 11.8
**Area of residence, N (%)**						
Northern Sweden	589 (23.7)	322 (22.9)	267 (24.8)	5,890 (23.7)	3,220 (22.9)	2,670 (24.8)
Central Sweden	1,330 (53.5)	762 (54.1)	568 (52.8)	13,300 (53.5)	7,620 (54.1)	5,680 (52.8)
Southern Sweden	565 (23.0)	324 (23.0)	241 (22.4)	5,650 (22.8)	3,240 (23.0)	2,410 (22.4)
PPI user (before index date), N (%)	704 (28.3)	372 (26.4)	332 (30.9)	5,961 (24.0)	3,072 (21.8)	2,889 (26.9)
cDDDs (mean ± SD)	534 ± 714	534 ± 730	534 ± 696	573 ± 769	583 ± 790	563 ± 746
PPI user (before index date), N (%)†	539 (21.7)	278 (19.7)	261 (24.3)	5,099 (20.5)	2,615 (18.6)	2,484 (23.1)
cDDDs (mean ± SD)†	515 ± 674	526 ± 686	505 ± 614	504 ± 674	517 ± 698	491 ± 647

*SD* standard deviation, *cDDDs* cumulative defined daily doses.

*Date of diagnosis for ALS patients and date of selection for controls.

^†^After inclusion of a lag window of 1 year.

**Table 2:** Association between PPI use and the risk of ALS, a nested case–control study in Sweden, 2006–2013.

Groups	Including a lag window of 1 year		Including a lag window of 2 years†		Including a lag window of 3 years†	
	Cases/controls	OR (95% CI)*	Cases/controls	OR (95% CI)*	Cases/controls	OR (95% CI)*
**Whole population**						
PPI non-user	1,945/19,744	1.00 (reference)	1,778/17,982	1.00 (reference)	1,545/15,623	1.00 (reference)
PPI user	539/5,096	1.08 (0.97–1.19)	426/4,058	1.06 (0.95–1.19)	331/3,137	1.07 (0.94–1.21)
**By cDDDs**						
Low tertile	179/1,811	1.01 (0.86–1.18)	127/1,366	0.94 (0.78–1.14)	103/1,068	0.98 (0.79–1.20)
Mid tertile	177/1,617	1.11 (0.95–1.31)	155/1,362	1.15 (0.97–1.37)	120/1,041	1.17 (0.96–1.42)
High tertile	183/1,668	1.12 (0.95–1.32)	144/1,330	1.10 (0.92–1.32)	108/1,028	1.07 (0.87–1.31)
p for trend		0.0874		0.1367		0.2309
**Male**						
PPI non-user	1,130/11,467	1.00 (reference)	1,035/10,396	1.00 (reference)	904/9,097	1.00 (reference)
PPI user	278/2,613	1.08 (0.94–1.25)	213/2,084	1.03 (0.88–1.20)	167/1,613	1.04 (0.87–1.24)
**By cDDDs**						
Low tertile	95/922	1.05 (0.84–1.31)	70/702	1.00 (0.78–1.29)	51/547	0.90 (0.70–1.26)
Mid tertile	93/821	1.15 (0.92–1.44)	68/689	0.99 (0.77–1.28)	56/530	1.06 (0.80–1.42)
High tertile	90/870	1.05 (0.84–1.33)	75/693	1.09 (0.85–1.40)	60/536	1.13 (0.85–1.50)
p for trend		0.3055		0.6117		0.4163
**Female**						
PPI non-user	815/8,277	1.00 (reference)	743/7,586	1.00 (reference)	641/6,526	1.00 (reference)
PPI user	261/2,483	1.07 (0.92–1.24)	213/1,974	1.11 (0.94–1.30)	164/1,524	1.10 (0.91–1.32)
**By cDDDs**						
Low tertile	84/889	0.96 (0.76–1.22)	57/664	0.88 (0.66–1.17)	52/521	1.02 (0.76–1.37)
Mid tertile	84/796	1.08 (0.85–1.37)	87/673	1.33 (1.05–1.68)	64/511	1.28 (0.97–1.68)
High tertile	93/798	1.19 (0.95–1.50)	69/637	1.11 (0.85–1.45)	48/492	1.00 (0.73–1.36)
p for trend		0.1603		0.1038		0.3766
**< 65 years**						
PPI non-user	733/7,508	1.00 (reference)	657/6,694	1.00 (reference)	561/5,672	1.00 (reference)
PPI user	148/1,302	1.17 (0.97–1.41)	111/986	1.15 (0.93–1.42)	79/728	1.10 (0.86–1.41)
**By cDDDs**						
Low tertile	59/563	1.08 (0.81–1.42)	45/404	1.14 (0.83–1.57)	38/305	1.26 (0.89–1.79)
Mid tertile	50/397	1.30 (0.95–1.76)	40/317	1.29 (0.92–1.81)	24/224	1.09 (0.70–1.67)
High tertile	39/342	1.17 (0.83–1.65)	26/265	1.00 (0.66–1.51)	17/199	0.86 (0.52–1.43)
p for trend		0.0919		0.3208		0.9064
**≥ 65 years**						
PPI non-user	1,212/12,236	1.00 (reference)	1,121/11,288	1.00 (reference)	984/9,951	1.00 (reference)
PPI user	391/3,794	1.04 (0.92–1.18)	315/3,072	1.03 (0.90–1.18)	252/2,409	1.06 (0.91–1.23)
**By cDDDs**						
Low tertile	120/1,248	0.97 (0.80–1.18)	82/962	0.86 (0.68–1.09)	65/763	0.86 (0.66–1.12)
Mid tertile	127/1,220	1.05 (0.87–1.28)	115/1,045	1.11 (0.91–1.36)	96/817	1.19 (0.95–1.49)
High tertile	144/1,326	1.10 (0.91–1.32)	118/1,065	1.12 (0.91–1.37)	91/829	1.11 (0.88–1.40)
p for trend		0.3145		0.2496		0.1953

*OR* odds ratio, *CI* confidence interval, *cDDDs* cumulative defined daily doses.

*Adjusted for age, sex, and area of residence.

^†^The application of longer lag windows resulted in lower case numbers because only cases diagnosed in later calendar years could have an observation period longer than the respective lag time of 2 and 3 years, respectively (2,204 cases and 22,040 matched controls using a lag window of 2 years and 1,876 cases and 18,760 controls using a lag window of 3 years).

## Discussion

In this nationwide nested case–control study from Sweden, we assessed for the first time the association between the use of PPIs and future ALS risk. Although ALS patients were more likely to use PPIs before diagnosis than controls, there was no association between PPI use and ALS risk when applying a lag window of 1, 2, or 3 years before diagnosis.

The importance of the utilisation of lag windows in pharmacoepidemiology has been emphasised in studies of dementia^19^, another proteinopathic neurodegenerative disease with a long pre-diagnostic disease course that shares pathomechanisms with ALS. Although earlier studies reported an association between PPI use and dementia risk^16,17^, a more recent nested case–control study based on nationwide health registers in Finland could not find a statistically significant association after applying lag windows of 1–5 years^20^. Lag windows are useful to assess the potential impact of reverse causation on the association between medication use and a disease risk, e.g. medications might be used to treat symptoms associated with an upcoming disease outcome^20^. Another reason for the application of lag windows is that ALS patients are more likely to consult a clinician after symptom onset (and before diagnosis) than controls^21^, and are therefore also more likely to be diagnosed with and treated for dyspepsia, which is very common in the general population with prevalence estimates over 20%^22^. Alternatively, as pain has been reported as one of the initial ALS symptoms^23^, PPIs could also have been prescribed together with nonsteroidal anti-inflammatory drugs (NSAIDs) to reduce NSAID-induced gastrointestinal adverse events^24^. A lag window of 1 year was therefore chosen in our study due to the well described diagnostic delay (i.e. time from symptom onset to diagnosis) of roughly the same length in ALS patients, which has been attributed to the insidious nature of the disease and referral from primary care to inappropriate specialists^25^. Similar results were obtained using lag windows of 2 or 3 years with no significant association between PPI use and ALS risk.

PPIs are among the most widely prescribed drugs worldwide, with prevalence estimates over 30% in the aged population^18^. Over-prescription and inappropriate use of PPIs have also been reported^26,27^. As a result, a risk increment of neurodegenerative diseases in relation to PPI use, if verified, might lead to important public health implications. More research with proper study design and analytical methodologies are clearly needed in further assessing the role of PPIs on neurodegenerative diseases, including not only dementia, Parkinson’s disease, but also ALS. In addition to its role on the risk of neurodegenerative diseases, the impact of PPIs on the progression of neurodegenerative diseases is also interesting. Although we did not find an association between PPI use and ALS risk in the present study, the role of PPIs on the survival of ALS patients needs to be studied further. A study of US veterans found an increased mortality among PPI users^28^, and a more recent study from Austria reported an increased mortality risk associated with PPI use among dementia patients and controls^29^. In terms of ALS, only one study examined the effect of various medications including PPIs on the disease course and failed to show a statistically significant relationship between PPIs and ALS survival after correction for multiple testing^30^. Future studies are clearly needed to address this question further.

Our study is characterised by the nationwide population-based design including virtually all ALS cases diagnosed during the study period in Sweden, as the Swedish health-care system covers the total population despite their socioeconomic status and age. Although we were not able to verify diagnoses for all patients with ALS, the Swedish Patient Register was shown to have a positive predictive value of > 90% for ALS diagnosis^31^. Another strength of our study was that exposure to PPIs was precisely quantified by the calculation of cDDDs, which was reported to accurately represent PPI exposure^32^. Our study has also limitations. Due to the lack of detailed information on clinical characteristics of ALS cases including site of onset, disease progression rate and family history, we were unable to assess whether the noted null association between PPI use and disease risk differed between ALS phenotypes. PPI use before July 1, 2005 was not captured by the Prescribed Drug Register and could therefore not be determined in this study, potentially leading to a misclassification of PPI exposure in ALS cases and controls to some extent. However, this bias is not likely to be of great magnitude as our study covered a sufficiently long total observation period of 8.5 years in the Prescribed Drug Register, which was longer than the covered period in comparable observational studies assessing the link between PPI use and dementia risk^16,17^. Over-the-counter use of PPIs was not captured by the Prescribed Drug Register, but as PPIs obtained over-the-counter are usually of smaller packages and at a much higher price per dose, the degree of misclassification is likely to be minor. Previous studies have also shown that prescription claims are a valid data source for assessing exposure to medications, even though some of the medications are available over-the-counter^33,34^. Moreover, not all ALS risk factors reported in literature could be accounted for as confounders in this study. ALS risk factors include family history, age, sex, smoking, body mass index (BMI), and lipid levels^2^. Among these, family history is unlikely to be a confounder, defined as a common cause for the exposure and the outcome (i.e. for PPI use and ALS risk), whereas smoking, BMI, and lipid levels might be considered as potential confounders. Because of the register-based nature of the present study, however, we could not identify information on these factors.

In summary, we found no association between PPI use and ALS risk when utilising a lag window of at least 1 year before diagnosis. The application of lag windows is important in pharmacoepidemiological studies on diseases with significant diagnostic delays.

## Methods

### Study design

The study base of this nested case–control study is the Swedish Total Population Register, which was cross-linked to the Swedish Patient Register, the Swedish Prescribed Drug Register, and the Causes of Death Register using the individually unique personal identity numbers assigned to all Swedish residents^35^. All individuals born in Sweden and alive on July 1, 2006 were included and followed from July 1, 2006 until (i) ALS diagnosis, (ii) emigration, (iii) death, or (iv) December 31, 2013, whichever came first. ALS diagnosis was ascertained from the Swedish Patient Register according to the 10th Swedish revision of the International Classification of Disease code G12.2^31,36^, with the date of the first related hospital visit—either as an outpatient or inpatient—defining the date of ALS diagnosis. Using the method of incidence density sampling^37^, we selected ten controls per ALS case on the date of ALS diagnosis. These controls were individually matched to the case by sex, age, and area of residence and had to be free of ALS on the diagnosis date of their corresponding cases. The date of ALS diagnosis in cases and the date of selection in controls were used as the index date for both cases and controls.

### PPI exposure

The Swedish Prescribed Drug Register contains information on the Anatomical Therapeutic Chemical (ATC) codes, dosages, and dates of prescriptions and dispenses of all drugs in Sweden since July 1, 2005^38^. In both cases and controls, information on all dispensed PPIs (ATC code A02BC) between July 1, 2005 and the index date was extracted from the Prescribed Drug Register. Because cases and controls were selected from July 1, 2006 onward, there was at least a 1-year observation period for all cases and controls to collect information on PPI use. Individuals that had at least one PPI dispense before the index date were classified as PPI users. The cumulative defined daily doses (cDDDs) for PPIs were calculated as the total dispensed number of defined daily doses between July 1, 2005 and the index date. PPI use was therefore defined first as a categorical variable with two groups (PPI user and PPI non-user) and then as three groups according to the tertile distribution of cDDDs. Different lag windows ranging between 1 and 3 years before the index date were applied to account for both reverse causation^20^ and the mean diagnostic delay of around 12 months in ALS patients in Sweden^39^; this means that PPIs used within a specific lag window were not considered in the respective analyses.

### Statistical analysis

Descriptive statistics were performed using means and standard deviations (SD). Conditional logistic regression models were fitted to estimate odds ratios (ORs) and their 95% confidence intervals (CIs) for ALS in relation to PPI use, because cases and controls were individually matched to each other. Analyses were performed after including lag windows of 1, 2 or 3 years before the index date, respectively. All analyses were repeated after stratification for sex (male or female) and age at the index date (< 65 years or ≥ 65 years). Data analyses were performed using SAS version 9.4 (SAS Institute Inc, Cary, NC). A two-sided p < 0.05 was considered statistically significant.

### Ethics approval and consent to participate

This study was approved by the Regional Ethical Review Board in Stockholm, Sweden (DNR: 2017/1985-32). Informed consent was not necessary due to the application of a register-based design and the use of pseudonymised patient information, and was waived by the Regional Ethical Review Board. All methods were performed in accordance with the relevant guidelines and regulations.

## Data Availability

Data are available for collaborative studies with qualified investigators upon request.

## References

[1] van Es MA (2017). Amyotrophic lateral sclerosis. Lancet.

[2] Ingre C, Roos PM, Piehl F, Kamel F, Fang F (2015). Risk factors for amyotrophic lateral sclerosis. Clin. Epidemiol..

[3] Ascherio A, Schwarzschild MA (2016). The epidemiology of Parkinson's disease: Risk factors and prevention. Lancet Neurol..

[4] Baumgart M (2015). Summary of the evidence on modifiable risk factors for cognitive decline and dementia: A population-based perspective. Alzheimers Dement..

[5] Dehay B (2013). Lysosomal impairment in Parkinson's disease. Mov. Disord..

[6] Majumdar A (2007). Activation of microglia acidifies lysosomes and leads to degradation of Alzheimer amyloid fibrils. Mol. Biol. Cell.

[7] Wolfe DM (2013). Autophagy failure in Alzheimer's disease and the role of defective lysosomal acidification. Eur. J. Neurosci..

[8] Cascella R (2017). Quantitative assessment of the degradation of aggregated TDP-43 mediated by the ubiquitin proteasome system and macroautophagy. FASEB J..

[9] Cipolat Mis MS, Brajkovic S, Frattini E, Di Fonzo A, Corti S (2016). Autophagy in motor neuron disease: Key pathogenetic mechanisms and therapeutic targets. Mol. Cell Neurosci..

[10] Koh JY, Kim HN, Hwang JJ, Kim YH, Park SE (2019). Lysosomal dysfunction in proteinopathic neurodegenerative disorders: Possible therapeutic roles of cAMP and zinc. Mol. Brain.

[11] Cheng FC, Ho YF, Hung LC, Chen CF, Tsai TH (2002). Determination and pharmacokinetic profile of omeprazole in rat blood, brain and bile by microdialysis and high-performance liquid chromatography. J. Chromatogr. A.

[12] Rojo LE, Alzate-Morales J, Saavedra IN, Davies P, Maccioni RB (2010). Selective interaction of lansoprazole and astemizole with tau polymers: Potential new clinical use in diagnosis of Alzheimer's disease. J. Alzheimers Dis..

[13] Colacurcio DJ, Nixon RA (2016). Disorders of lysosomal acidification-The emerging role of v-ATPase in aging and neurodegenerative disease. Ageing Res. Rev..

[14] Liu W (2013). Inhibition of lysosomal enzyme activities by proton pump inhibitors. J. Gastroenterol..

[15] Nielsen HH, Qiu J, Friis S, Wermuth L, Ritz B (2012). Treatment for Helicobacter pylori infection and risk of Parkinson's disease in Denmark. Eur. J. Neurol..

[16] Gomm W (2016). Association of proton pump inhibitors with risk of Dementia: A pharmacoepidemiological claims data analysis. JAMA Neurol..

[17] Haenisch B (2015). Risk of dementia in elderly patients with the use of proton pump inhibitors. Eur. Arch. Psychiatry Clin. Neurosci..

[18] Halfdanarson OO (2018). Proton-pump inhibitors among adults: A nationwide drug-utilization study. Therap. Adv. Gastroenterol..

[19] Moayyedi P, Lewis MA (2017). Proton pump inhibitors and dementia: deciphering the data. Am. J. Gastroenterol..

[20] Taipale H (2017). No association between proton pump inhibitor use and risk of Alzheimer's disease. Am. J. Gastroenterol..

[21] Galvin M (2017). The path to specialist multidisciplinary care in amyotrophic lateral sclerosis: A population-based study of consultations, interventions and costs. PLoS ONE.

[22] Ford AC, Marwaha A, Sood R, Moayyedi P (2015). Global prevalence of, and risk factors for, uninvestigated dyspepsia: A meta-analysis. Gut.

[23] Chio A, Mora G, Lauria G (2017). Pain in amyotrophic lateral sclerosis. Lancet Neurol..

[24] Gwee KA, Goh V, Lima G, Setia S (2018). Coprescribing proton-pump inhibitors with nonsteroidal anti-inflammatory drugs: Risks versus benefits. J. Pain Res..

[25] Turner MR, Talbot K (2013). Mimics and chameleons in motor neurone disease. Pract. Neurol..

[26] Forgacs I, Loganayagam A (2008). Overprescribing proton pump inhibitors. BMJ.

[27] Heidelbaugh JJ, Kim AH, Chang R, Walker PC (2012). Overutilization of proton-pump inhibitors: What the clinician needs to know. Therap Adv. Gastroenterol..

[28] Xie Y (2017). Risk of death among users of Proton Pump Inhibitors: A longitudinal observational cohort study of United States veterans. BMJ Open.

[29] Cetin H (2020). Increased risk of death associated with the use of proton-pump inhibitors in patients with dementia and controls—a pharmacoepidemiological claims data analysis. Eur. J. Neurol..

[30] Cetin H (2015). Associations between co-medications and survival in ALS-a cohort study from Austria. J. Neurol..

[31] Mariosa D (2017). Blood biomarkers of carbohydrate, lipid, and apolipoprotein metabolisms and risk of amyotrophic lateral sclerosis: A more than 20-year follow-up of the Swedish AMORIS cohort. Ann. Neurol..

[32] Sinnott SJ, Polinski JM, Byrne S, Gagne JJ (2016). Measuring drug exposure: concordance between defined daily dose and days' supply depended on drug class. J. Clin. Epidemiol..

[33] Holmes HM (2009). Rational prescribing for patients with a reduced life expectancy. Clin. Pharmacol. Ther..

[34] Yood MU (2007). Using prescription claims data for drugs available over-the-counter (OTC). Pharmacoepidemiol. Drug Saf..

[35] Ludvigsson JF, Otterblad-Olausson P, Pettersson BU, Ekbom A (2009). The Swedish personal identity number: Possibilities and pitfalls in healthcare and medical research. Eur. J. Epidemiol..

[36] Ludvigsson JF (2011). External review and validation of the Swedish national inpatient register. BMC Public Health.

[37] Lubin JH, Gail MH (1984). Biased selection of controls for case–control analyses of cohort studies. Biometrics.

[38] Wettermark B (2007). The new Swedish Prescribed Drug Register–opportunities for pharmacoepidemiological research and experience from the first six months. Pharmacoepidemiol. Drug Saf..

[39] Longinetti E (2018). The Swedish motor neuron disease quality registry. Amyotroph. Lateral Scler. Frontotemporal. Degener..

